# Bilateral Ovarian Vein Thrombosis in a Post-menopausal Woman Leading to Diagnosis of Non-gynecological Malignancy: A Case Report

**DOI:** 10.7759/cureus.97312

**Published:** 2025-11-20

**Authors:** Ariba Mumtaz, Muhammad Nasser Javaid, Bisma Nazir, Mohamad Hasan, Yasir Ihsan

**Affiliations:** 1 Respiratory Medicine, Blackpool Victoria Hospital, Blackpool Teaching Hospitals NHS Foundation Trust, Blackpool, GBR; 2 Pulmonology and Critical Care, Blackpool Victoria Hospital, Blackpool Teaching Hospitals NHS Foundation Trust, Blackpool, GBR; 3 Radiology, Blackpool Victoria Hospital, Blackpool Teaching Hospitals NHS Foundation Trust, Blackpool, GBR; 4 Diabetes and Endocrinology, Royal Lancaster Infirmary, Lancaster, GBR

**Keywords:** anticoagulation, imaging, malignancy, ovarian vein thrombosis, post-menopausal

## Abstract

Bilateral ovarian vein thrombosis (OVT) is hard to find, particularly in post-menopausal women. We report the case of a 62-year-old woman who presented with abdominal pain and was found to have bilateral OVT secondary to adenocarcinoma of the sigmoid colon. This case illustrates OVT as a potential sentinel sign of underlying malignancy and highlights the importance of reviewing incidental radiological findings. Early recognition and multidisciplinary management are crucial to prevent complications and enable timely diagnosis and treatment.

## Introduction

Ovarian vein thrombosis (OVT) is a type of atypical venous thromboembolism (VTE), and while it's uncommon, it remains a potentially life-threatening condition that is most frequently associated with pregnancy, pelvic infection, oral contraceptives, malignancy, or recent surgery; however, some cases can be idiopathic [1]. It occurs in approximately 0.01-0.02% of all vaginal deliveries and 0.1 % of cesarian births, with the right ovarian vein being predominantly affected due to anatomical and physiological factors [2]. Diagnosis is generally established via conventional radiological techniques, including contrast-enhanced CT or MRI [3].

While there is limited argument surrounding spontaneous resolution of OVT without treatment [4], the common consensus is to treat with anti-coagulants to prevent complications such as pulmonary embolism, Inferior vena cava thrombosis; however, the choice of anti-coagulants and duration of treatment remains situation specific [5].

This case report attempts to present an unusual finding of bilateral OVT in a post-menopausal woman, which ultimately led to the diagnosis of a new underlying gastrointestinal malignancy. This case highlights the importance of maintaining a high index of suspicion for OVT in females of any age group, especially post-menopausal women who present with vague abdominal pain. It also highlights the importance of thorough evaluation to identify potentially serious underlying systemic causes such as malignancy.

## Case presentation

A 62-year-old post-menopausal lady was admitted to the hospital in September 2025 after referral from her general practitioner (GP). She had a few days' history of mild lower abdominal pain and per-rectal bleeding. The patient had a background of hypertension, osteoporosis, and mild chronic obstructive pulmonary disease and was taking ramipril 5 mg once daily, a long-acting beta-agonist (LABA)/long-acting muscarinic antagonist (LAMA) combination inhaler, salbutamol inhaler, and calcium and vitamin D supplements. She had never smoked, and there was no history of recent/past general/pelvic surgeries or infections. Her last childbirth via cesarean section was 38 years ago. She had no past medical history of miscarriages or any thromboembolic episodes, and neither was there any family history of malignancies or thrombotic disorders.

On initial examination, she had mild lower abdominal tenderness, but there was no guarding or palpable masses. Per-rectal exam was unremarkable with no significant findings such as lumps/ulcer/hemorrhoids/skin tears. Initial Laboratory investigations showed a normal total leucocytic count and raised D-dimer. Hemoglobin levels were 63 g/dl (reference range 115-158 g/dl), normochromic, normocytic. She was transfused with two units of packed RBCs with post-transfusion hemoglobin of 84 g/dl. Results of initial relevant laboratory investigations are summarised in Table 1. 

**Table 1:** Initial laboratory findings CEA: carcinoembryonic antigen; CRP: C-reactive protien; CA: cancer antigen

Parameter	Patient value	Reference range	Units	Remarks
Hemoglobin	63	118-158	g/L	Low
White blood cells	6.6	3.60-10.5	x10^9/L	Normal
Platelets	303	150-450	x10^9/L	Normal
CA 125	10	0-35	IU/ml	Normal
CEA	3	0-5	ug/L	Normal
CA 19-9	<1.2	Less than 37	IU/ml	Normal
CRP	7	0.2-4.9	mg/L	High
D-dimer	980	0-500	ng/L	High

**Figure 1:**
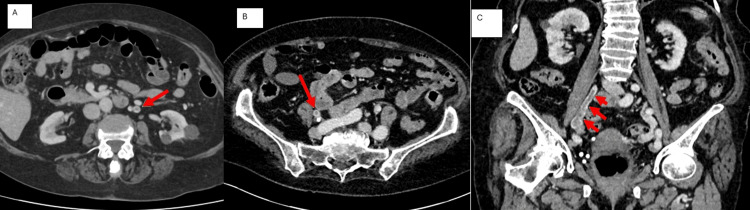
CT scan of the abdomen and pelvis with contrast (axial view) showing (A) left ovarian vein thrombosis (red arrow) and (B) right ovarian vein thrombosis (red arrow). (C) CT scan of the abdomen and pelvis with contrast (coronal view) showing right ovarian vein thrombosis (red arrow)

**Figure 2:**
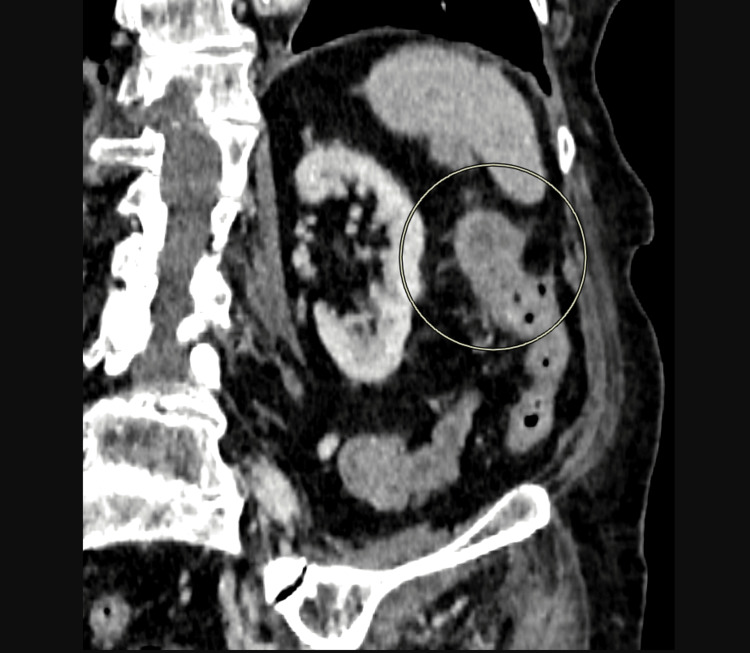
CT scan finding of thickening of sigmoid colon as marked by circle

For further workup, contrast-enhanced CT of the abdomen and pelvis was arranged, which revealed filling defects and mural enhancement within the ovarian veins bilaterally, suggestive of OVT and thickening of the sigmoid colon (Figures 1, 2).

The clinical context of low hemoglobin and CT scan finding of sigmoid thickening warranted further investigation, which is why flexible sigmoidoscopy + biopsy was undertaken, leading to a new confirmed diagnosis of adenocarcinoma of sigmoid colon. We discussed this with the on-board gynecology team, who suggested referral to colorectal multidisciplinary team (MDT) and hematology for further treatment, with no need for further gynaecological input. 

The patient was thus promptly referred to the colorectal MDT, who, in liaison with oncology, started the patient on palliative chemotherapy, andthe patient has recently received her first cycle of chemotherapy under their joint care. The patient was also commenced on enoxaparin as a treatment for bilateral OVT after consulting hematology. The decision to continue anti-coagulation for the long term is yet to be decided by oncology, based on the likelihood of whether the underlying malignancy is treatable or not. 

## Discussion

This report describes the case of a 62-year-old post-menopausal woman who presented with bilateral OVT. Studies have shown that pregnancy-related OVT is mostly unilateral (right-sided), citing 70-90% prevalence, and bilateral cases are quite scarce (11-14%) [6]. However, the prevalence of unilateral vs bilateral OVT in non-pregnant women with cancer is not well established. 

OVT has been found linked to various conditions such as malignancy, hypercoagulable states, inflammatory diseases, and occasionally idiopathic cases [7]. Common malignancies linked with OVT include breast cancer [8], pancreatic cancer [9], but ovarian cancer remains one of the most common gynecological cancers to be linked with venous thromboembolism in general [10]. While gynecological malignancy, such as breast or ovarian cancer, is an established risk factor, OVT has not been seen in patients with colon cancer, which makes our case unique, as our patient had OVT with adenocarcinoma of the sigmoid colon. 

Malignancy in general is a well-recognized prothrombotic condition that can lead to venous thrombosis at unusual sites, including the ovarian veins [11]. The underlying mechanism can be explained by Trousseau’s syndrome, which is a paraneoplastic hypercoagulable state in which thrombosis serves as an early sign of occult malignancy [12]. The pathophysiology aligns with Virchow’s triad, as tumors promote hypercoagulability through the release of cytokines and tissue factors, cause venous stasis via extrinsic compression, and induce endothelial injury through direct invasion or local inflammation [13]. These mechanisms create an environment propitious to thrombosis in both typical and atypical venous locations. It’s important to recognize that while common thromboses may arise from well-known risk factors, thrombosis at unusual sites, such as OVT, remains uncommon and should prompt further investigations into hidden cancer 

Our patient presented with vague abdominal pain and per-rectal bleeding, while patients with OVT generally present with fever, abdominal/ pelvic pain, or abdominal mass. However, some asymptomatic cases have been reported, leading to OVT sometimes being picked up incidentally on screening investigations [14]. 

Our patient had a CT abdomen-pelvis with contrast, which led to the diagnosis of bilateral OVT, and this modality of imaging continues to be popular for diagnosing OVT. While CT with contrast has 78% sensitivity and 62% specificity, magnetic resonance angiogram has been found to have 100% specificity and 92% sensitivity; however, CT takes precedence over MRI due to ease of availability [15]. Furthermore, early recognition and appropriate management of OVT are essential to prevent serious complications such as thrombus extension into the inferior vena cava or pulmonary embolism [16] or septic thrombophlebitis [7]. 

Treatment generally involves initiation of anticoagulants and definitive management of the underlying cause, such as cancer in our patient. Low molecular weight heparin (LMWH) or direct oral anticoagulants (DOACs) are commonly used [17]. The optimal duration of anti-coagulation is not standardized but remains like lower limb deep venous thrombosis (DVT), and it generally ranges from three to six months or until the provoking factor(s) such as malignancy have been resolved [18]. In malignancy-associated thrombosis, extended anticoagulation may be warranted and is situation-specific. Our patient was started on LMWH (enoxaparin) for anticoagulation after prompt discussion with hematology, and the initial consensus was to continue enoxaparin for six months until the patient was seen by the oncology team, who would decide further anticoagulation based on cancer response to chemotherapy and disease remission, recurrence, and progression. 

There was a discussion in the colorectal MDT about putting an inferior vena cava (IVC) filter for prevention of propagation of the OVT clot; however, while there has been some debate on use of IVC filters for improving prognosis of patients with OVT and gynecological cancer, a metanalysis found no evidence between use of IVC filters and improved survival outcome in the general population [19]. 

**Figure 3:**
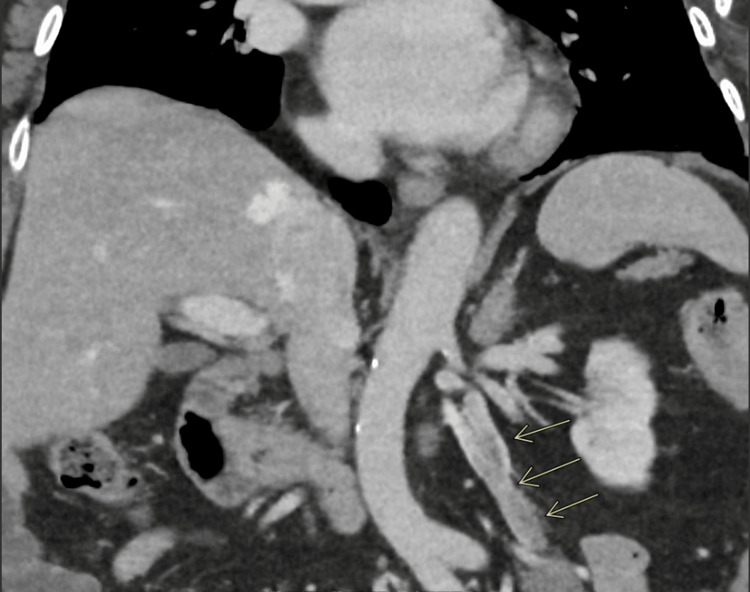
CT scan of abdomen with contrast (coronal view), showing left ovarian vein thrombosis (arrows)

This case was also discussed in our local radiology MDT, and an incidental key learning point was established. While comparing the patient’s recent CT scan to a CT scan performed two years earlier, it was found that the scan two years ago did show left-sided OVT, which was not reported at that time (Figure 3). This represents a classic “look-back” phenomenon in radiology, underscoring the importance of recognizing and following up incidental findings, as it's common in busy clinical settings to overlook small or atypical vascular findings.

While our patient continues to follow up with oncology and colorectal MDTs, OVT generally bears a good prognosis if prompt and appropriate treatment is initiated. A study has shown that while the five-year mortality rate of OVT patients is 43% compared to 20% in DVT; however, all OVT deaths were cancer-related. Hence poor survival rate in OVT seems to be directly linked to the presence of underlying malignancy. Recurrence rates are generally low and were found to be comparable to lower limb DVT [18].

## Conclusions

Our patient is a post-menopausal woman diagnosed with bilateral OVT linked with a new sigmoid adenocarcinoma, and highlights the importance of considering OVT as a potential differential for patients presenting with abdominal pain in the clinical setting. It also reflects that while OVT remains uncommon, prompt diagnosis and treatment can prevent complications; however, duration and choice of anticoagulant remain unclear and situation-specific. While recurrence risks are low, the prognosis of OVT continues to be graver than lower limb DVT and mainly rests on the curability of underlying causative conditions like cancer.

We also reflect on some key lessons learnt: first, OVT or any atypical venous thrombosis can and should serve as a sentinel event prompting further investigation to exclude underlying occult malignancy. Second, incidental vascular findings on prior imaging should warrant careful review and follow-up, and third, early recognition and multidisciplinary management are vital to improving outcomes and preventing complications. 
